# Voices from the Youth in Kenya Addressing Mental Health Gaps and Recommendations

**DOI:** 10.3390/ijerph19095366

**Published:** 2022-04-28

**Authors:** Peter Memiah, Fernando A. Wagner, Robert Kimathi, Naomi Idah Anyango, Samuel Kiogora, Stella Waruinge, Faith Kiruthi, Shillah Mwavua, Celina Kithinji, Jeremiah Okuto Agache, Wincolyne Mangwana, Niyibeshaho Marie Merci, Leonidah Ayuma, Samuel Muhula, Yvonne Opanga, Maureen Nyambura, Annrita Ikahu, Lillian Otiso

**Affiliations:** 1 Division of Epidemiology and Prevention, Institute of Human Virology, University of Maryland Baltimore, Baltimore, MD 21201, USA; 2 School of Social Work, University of Maryland Baltimore, Baltimore, MD 21201, USA; fernando.wagner@ssw.umaryland.edu; 3 LVCT Health, Nairobi P.O. Box 19835-00202, Kenya; robert.kimathi@lvcthealth.org (R.K.); annrita.ikahu@lvcthealth.org (A.I.); lilian.otiso@lvcthealth.org (L.O.); 4 Ministry of Health Kenya, Nairobi P.O. Box 30016-00100, Kenya; anyangonomy@yahoo.com (N.I.A.); samkiogo63@gmail.com (S.K.); maureen.nyambura@gmail.com (M.N.); 5 Nairobi Metropolitan Services, Nairobi P.O. Box 30430-00100, Kenya; swaruinge15@gmail.com (S.W.); faykiruthi@gmail.com (F.K.); smwaniga@yahoo.com (S.M.); 6 Mombasa County Department of Health, Mombasa P.O. Box 81599-80100, Kenya; kithinjicl@gmail.com; 7 Kisumu County Department of Health, Kisumu P.O. Box 721-40100, Kenya; jeremiahokuto2@gmail.com; 8 Youth Advisory Champions for Health, Nairobi Youth Advisory Council, Mombasa P.O. Box 81599-80100, Kenya; wincolynemangwana@gmail.com (W.M.); mariemerci68@gmail.com (N.M.M.); leuayuma@gmail.com (L.A.); 9 Amref Health Africa in Kenya, Nairobi P.O. Box 30125-00100, Kenya; samuel.muhula@amref.org (S.M.); yvonne.opanga@amref.org (Y.O.)

**Keywords:** mental health, adolescents and young people, digital health, Youth Advisory Champions for Health (YACH), Ministry of Health (MoH), participatory research

## Abstract

Studies including adolescents and young people (AYP) enhance the relevance of research results, benefit stakeholders, and inform future research. There exists a mental health gap in services for AYP living in low and middle-income countries. This study aims to identify mental health challenges faced by adolescents and young people in Kenya, develop practical recommendations to mitigate these issues, and reduce the mental health burden among this population. We convened an AYP-led meeting that involved 41 participants. The meeting objectives were to (1) identify efforts to support existing national and regional strategic priorities and review goals for addressing mental health needs among AYPs, (2) develop immediate action plans for strengthened mental health services, (3) review and strengthen country-level coordination mechanisms, and (4) identify how participating county experiences can inform mental health services in Kenya. Ministry of Health (MoH) officials from national and county levels, academic experts, and implementing partner agencies involved in mental health services participated in the meeting. The team, including AYP representatives, identified various mental health challenges among the AYA and recommended interventions aimed towards improving their mental health situation in the country. The challenges were clustered into three themes and comprehensively reviewed to establish the precipitating factors to mental health outcomes among AYPs in Kenya and provide recommendations. The themes included (1) legislative, (2) service provider/Ministry of Health, and (3) adolescent/individual-level factors. To bridge the mental health gap in the country and scale up mental health outcomes, the stakeholders recommended interventions within the context of the three clusters. The key suggestions included an increase in insurance financing, acceleration of community health interventions, the establishment of adolescent-friendly spaces, the training of adolescent youth champions, interactive service provision models, implementation of the existing mental health policies and structures, the development of comprehensive assessment tools, well equipped mental health departments in health facilities, the enhancement of telehealth services and digital villages, the mobilization of a functional mental health response team, and the development of a mental health database.

## 1. Introduction

Adolescence is a period associated with several changes, and multi-dimensional factors impact mental health outcomes at this age [1]. According to the World Health Organization Atlas (WHO), the mental health outcomes of this population are directly proportional to the intensity of risks that adolescents are exposed to during this developmental stage. The WHO Atlas also indicates a hurdle in offering mental health services globally and inequalities in allocating mental health resources, which compromises the access to and quality of mental health services, especially in low and middle-income countries. Globally, mental health issues affect 10–20% of AYP, and the onset age for 50% of these issues is 14 years [2]. Mental health disorders also contribute to 16% of the disease load among AYPs [3].

Data on the prevalence of mental health, neurological issues, and substance use (MNS) in Kenya are limited [4]. The Kenyan National Commission of Human Rights estimates that 25% and 40% of outpatients and inpatients suffer from mental health conditions. The most frequent diagnoses of mental illnesses made in general hospital settings are depression, substance abuse, stress, and anxiety disorders [5]. A WHO report (2017), ranked Kenya fifth among African countries with elevated depression cases, with global statistics indicating that approximately two million people suffer from depression [6,7]. Such mental health conditions continue to accelerate rapidly, with approximately one of each four Kenyans presenting with a mental health disorder at one point in their lives [8]). Additionally, the World Population Review places Kenya at 114 of 175 countries with escalated suicidal rates (6.5 per 100,000 persons) [9]. Other mental health issues affecting youth include anxiety, conduct disorders, attention-deficit/hyperactivity disorders (ADHD), and personality disorders that could potentially affect quality of life [10]. Some of the factors associated with increased mental health among this cadre include anxiety, drug and substance disorders, aggression, depression, sexual and gender-based violence (SGBV), and self-harm. The COVID 19 pandemic exacerbated mental health issues in the country, pushing the President to declare mental health a national priority [8].

The HIV burden in sub-Saharan Africa (SSA) equally affects adolescents’ mental health during this transition period [11]. It is evidenced that people living with HIV (PLHIV) report higher rates of mental health issues, including stress, depression, anxiety, and trauma. Data collected from various heterogeneous studies conducted in this region over the last decade indicate that up to 25% of adolescents suffer from neurological conditions and up to 50% manifest behavioral and emotional disorders. Although few studies have focused on the nexus between HIV and mental health, there is an increasing awareness of the broader HIV epidemic’s enormous mental health burden [12,13,14].

AYPs’ mental health care requirements are often unaddressed, leading to a treatment gap due to a poor care-seeking attitude [15]. Challenges of managing mental conditions in Kenya include low awareness, limited treatment options, and implied costs of treating mental illnesses [16,17,18]. Significant data gaps exist regarding the implication of mental cases in low and middle-income countries such as Kenya, making it difficult to mitigate the pandemic. More information about access and utilization of mental health services is critical in improving mental health outcomes among the AYP.

The Kenya Mental Health Policy (2015–2030) [19] provides a framework that guides mental health reforms in the country with the aim of ensuring that all persons have access to comprehensive, integrated, and high-quality mental health care that is promotive, preventive, curative, and rehabilitative at all levels of healthcare. The policy also highlights vital strategies in mitigating the structural challenges and emerging patterns in alleviating mental health problems and disorders. Despite the availability of mental health policies and the country’s focus on addressing the gaps, Kenya has an increasing (AYP) population with undocumented mental health needs hampering resource allocation and service provision [20]. Like other SSA countries, Kenya struggles with legislative issues that inhibit the potent implementation of the existing mental health policies leading to the continually widening mental health treatment gap [21,22]. This paper highlights adolescent-led stakeholder recommendations with the aim of improving mental health among AYP in Kenya. The study objective supports the Kenya Mental Health policy (2015–2030). It creates an empirical baseline for outlining and establishing culturally appropriate and population-focused directions, interventions, and models applicable by schools, families, healthcare providers, and societies to address mental health among AYP.

## 2. Methods

### 2.1. Setting and Participants

The paper presents findings from an inception meeting with relevant stakeholders as one of the activities anchored on a cross-sectional survey that utilized mixed methods of data collection. The design entailed using an innovative multimodal approach to data collection, including a desk review of reports and data from the Mental Health Counselling Call Center, a stakeholder inception meeting with AYA leaders and key stakeholders in mental health in Kenya, and a phone-based survey using the REACH AYA App. The stakeholder inception meeting adopted a qualitative study design using focused panel discussions to establish mental health needs and risk factors, including individual, community, and health systemic factors influencing mental health among adolescents and young adults in three selected counties in Kenya.

The joint inception meeting targeted 41 participants, including key stakeholders who intervene in mental health and adolescents and young persons. The study deliberately selected AYP >18 years or mature minors who consented to be part of the study; who resided in the three selected urban counties in Kenya, including Nairobi, Kisumu and Mombasa counties; and who were adolescent leaders from youth councils. In addition, Youth Advisory Champions for Health (YACH) county representatives were included. Those who did not consent were excluded. The YACH represent over 25,000 youth in their catchment area. With regards to other stakeholders, the study included representatives from the Ministry of Health and Youth Affairs at the county level, representatives from NGOs implementing mental health programs in selected counties, mental health experts, and community health volunteers. The research and program implementers were participants directly addressing/supporting youth mental health needs in Kenya or were involved in similar research. Of the 41 participants of the stakeholder inception meeting, 39% were male and 61% female. In total, 16 youth and 25 other stakeholders were present, as indicated in Table 1.

The three counties were selected because of the high burden of mental health issues reported by adolescents through the call center. These three counties in urban settings are characterized by congestion, high levels of unemployment, inadequate social services, poverty, insecurity, crime, and hopelessness. This forms a unique backdrop in which to study AYA mental health issues.

Nairobi County is characterized by large intra-urban variations in educational attainment. Unemployment and limited livelihood opportunities are associated with an increased risk of involvement in anti-social behaviors, drug abuse, risky sexual behaviors, and a higher likelihood of dropping out of school among young people in Nairobi as a result of mental health issues experienced. During the transition into adulthood, AYP become increasingly more reliant on peers and less on parents and caregivers. While some successfully navigate these challenges, many require additional support, with peaking rates of accidental motorcycle deaths, alcohol use, suicide, mental health concerns, low self-esteem, substance use, HIV, and sexually transmitted infections.

Kisumu County is predominantly a peri-urban county situated in the Nyanza region of Kenya near Lake Victoria. The county reports the highest rate of teenage pregnancy in Kenya (22.2%) and high rates of domestic violence (10.3% of men and 49.5% of women report domestic violence in Nyanza compared to 8.6% of men and 38.4% of women in Kenya as a whole). Nyanza also has a considerably higher prevalence of HIV than the rest of Kenya, at 17.7% in women and 14.1% in men. All these socioeconomic and health challenges may impact the mental health of the population. Fishing was a key occupation around Lake Victoria, but the invasion of the lake by water hyacinth has reduced fishing and aggravated poverty and food insecurity. Thus, people living in Nyanza are grappling with poverty and the highest rates of HIV, malaria, and common childhood diarrheal illnesses in the country and are therefore facing high rates of life-threatening conditions and high levels of domestic violence, especially women.

Mombasa County is an urban county in which one of the three major cities of Kenya (Mombasa) is located, and it has an estimated population of 1.2 million people. Mombasa is the second-largest city in Kenya. Because of urbanicity, the county consists of a mix of local and immigrant communities from other parts of Kenya. The main economic activity in this county is tourism, which contributes about 68% of the employment wage. HIV prevalence among individuals aged 15 years and above in this county is estimated at 7.5%—1.2 times higher than the national prevalence. Urbanization in developing countries is associated with changes in social support and life stress, which are known to have an adverse effect on mental health.

### 2.2. Activities

A one-day (five-hour) stakeholder meeting was conducted on 19 July 2021 to understand the mental health situation in Kenya, including the mental health needs of the AYP in the study and gather input for interventions. The meeting adopted a blended approach where most stakeholders joined the meeting virtually via zoom. A panel discussion guide was utilized incorporating Normalization Process Theory (NPT) techniques which provided a framework for understanding and evaluating the processes for information collection toward implementation. This was done by identifying moot points for collective action in routinely incorporating complex interventions such as avenues towards mental health integration into everyday practice. 

The sessions were audio recorded for purposes of transcription and were led by a moderator and note taker. The research and program implementers were participants who were directly addressing/supporting youth mental health needs in Kenya or were involved in smilar research.

Ethical approval for the study was sought from the University of Maryland Ethical Committee, Amref Health Africa Ethics and Scientific review committee (Protocol Number P983-2021), and a Research Permit was obtained from National Commission of Science, Technology and Innovation. Key ethical considerations were met to ensure the maximum protection of study participants and study staff. Written informed consent was sought from participants before the meeting started. Consent for audio recording was sought from all participants before the meeting. All participants were aged >18 years. The meeting was attended by Youth Leaders from Youth Council and Youth Advisory Champions for Health. This group of Youth Leaders mainly play advocacy roles in pushing the health agenda for the youth. They work closely with county and national government, civil society organizations, and development partners in pushing for youth agenda in health. Therefore, there were minimal power differentials during the discussions, and Youth Leaders were open and clearly articulated the issues facing the youth without fear.

Several strategies were used to protect participant confidentiality. All survey data were encrypted, thus maintaining the confidentiality of responses. Data were stored on a secure system and de-identified before storage. There was no risk for participation in the study. A counselor was available from the team of key stakeholders, and any youths who required psychosocial support were provided with this. All measures to protect participants from COVID 19 were observed, including providing adequate PPE and masks and the observation of physical/social distancing during the meeting for those who attended physically. Most of the participants, however, joined online.

### 2.3. Data Processing and Analysis

After the audio recording, the data were uploaded through password-encrypted laptops into the University of Maryland workspace server. The workspace server is a private database that is encrypted and password protected. Communication between the browser and the server was encrypted using Secure Sockets Layer (SSL). Servers hosting the system were secured by firewalls to prevent unauthorized access and distributed denial of service attacks (DDOS). The audio recordings for FGDs were transcribed verbatim.

Data were analyzed using the content analysis approach, which entailed multiple readings of the transcripts to capture context and meaning, followed by coding and categorizing recurring concepts and ideas. Two trained independent researchers for text element and keyword coding reviewed the transcripts separately. A coding matrix of all categories of overarching themes was developed, and codes were compared and added or removed based on the agreement between all stakeholders. After coding, the emerging themes were organized based on the meeting objective. Recurring themes that emerged were noted and served to validate data obtained from the discussions. The data content was then analyzed manually and digitally using NVIVO (version 11, QSR International), and consensus was reached on the central and specific emergent themes.

Through this, the evaluation shifted from descriptive to a more interpretive thematic analysis. The process involved reviewing the transcript for wider topics and broad themes and translating the information into specific themes. The consensus was then analyzed and built up by a team of specialists (n = 12) who were carefully chosen to balance experience and professionalism in mental health areas and enhance multiplicity in themes and perspectives in the document. When available, the team applied for systematic reviews as the predominant literature sources (see below)—the team communicated through continuous emails, conference calls, and reviews. The statements were sorted and reframed for clarity by the study participants. Therefore, the views were edited for quick comprehension and relevance to the mental health context, eliminating redundant and irrelevant responses. An initial version of this statement was drafted and subsequently shared with panel members from this background work. The final version of the consensus article was created based on feedback and contributions from all participating members.

## 3. Results

This section highlights the mental health challenges and recommendations made by stakeholders to address the identified challenges.

**Figure 1:**
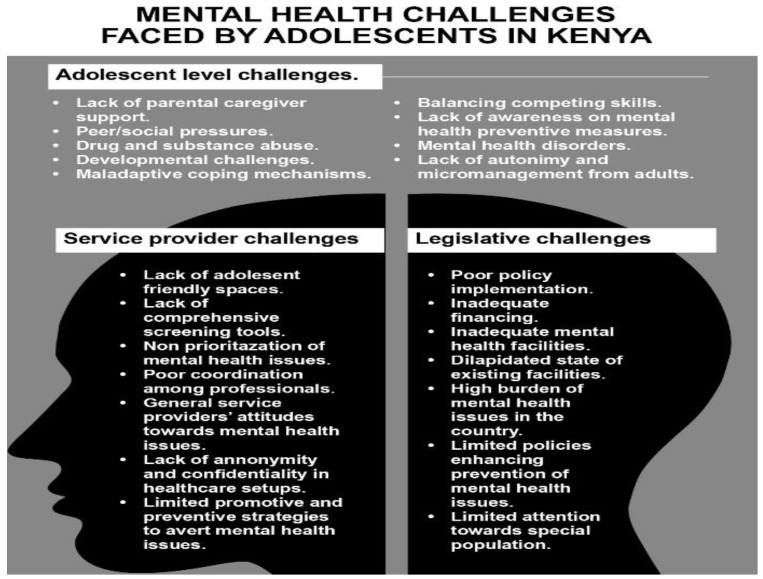
The image illustrates the adolescent mental health challenges identified by the adolescents.

The stakeholders categorized the mental health challenges faced by AYP in Kenya into three main clusters—legislative, service provider, and adolescent-level issues—as indicated in Figure 1 below.

### 3.1. Legislative Mental Health Gaps

The stakeholders highlighted the legislative gaps affecting adolescent mental health in the country. One of the key challenges included limited attention to special populations, including adolescents and young adults.

“*….then also, there was a mental health gap among special populations, whereby it was found that there are more people affected by the burden of ill mental health. This vicious cycle leads to a high impact on their quality of life. Vulnerable and special populations that were identified by the task force were the youth, elderly, the prisoners, the disciplinary forces, boy child, refugees, and so on…*”

MoH representative.

The identified legislative and structural gaps included compromised coordination mechanisms within the county and national government structures that affect service delivery and hamper mental health service reforms. Additionally, slow policy implementation and inadequate financing were evident barriers to mental health service delivery.

“*…Another finding was that, in terms of leadership and governance, there are inadequate mental health structures and leadership structures both at national and county levels, the Kenya Board of Mental Health is not constituted, and there are no mental coordination mechanisms or focal points in health management teams in most counties. We have actually just concluded an activity that we were doing to determine the mental health resources in the country in all the counties, and if you look at the numbers that we have, they are not so great…*”

MoH Representative.

Limited mental health infrastructure and or unfriendly mental health structures within the country were also documented to hamper access to mental health services.

“*We have about 44 facilities that can offer mental health services against the backdrop of more than four million people; this is an extra populated number according to the World Health Organization of people who could be suffering from mental illness. If you compare that number, clearly it shows that it doesn’t tally so well.*”

MoH Representative.

Inadequate preventive and promotional programs were identified to accelerate the high mental health burden, with most efforts focusing on mitigating existing mental health disorders while disregarding the causal factors.

“*…..Objective two is to implement programs for promotional mental health and prevention, and the action would include multi-sectoral health promotion programs in schools, communities, and workplaces; this means having to function multi-sectorial mental health promotion programs in all the counties by 2023, then mental health literacy and stigma reduction, which means a curriculum that can be given to, for example, teachers right from primary all the way to campus so that they can be able to help the students that they deal with. Then, there is a multi-sectoral preventive program, and this includes suicide prevention and substance use and prevention control so that we can reduce suicide mortality by 10% and also reduce alcohol consumption by 10%...*”

MoH representative.

### 3.2. Service Provider Mental Health Gaps

The AYP reported a gap in the number of trained service providers who can handle mental health issues among AYP in the few public facilities offering mental health services.

“*Public facilities do not have trained mental health personnel. They focus on other areas, but they are only trained in a certain line, so they are reluctant…*”

Adolescent representative.

Negative mental health narratives and general health care provider attitudes towards mental health issues also pose hurdles for AYP seeking mental health care, mainly due to lack of access to youth-friendly services. The age difference between the healthcare provider and adolescents contributed to the negative attitudes, leading to fear and bias in services provided.

“*Imagine going somewhere where you will be judged wrongly or somewhere where you will be overjudged… For instance, if a young person walks into a facility where there is an older woman, maybe their mom’s or dad’s age, they might find it difficult to access these services because of the age gap and as the social norm, maybe it expects us to behave in a straight way, imagine walking in a facility and maybe telling an older woman that maybe you are gay or lesbian or maybe you belong to LGBTQ community.*”

Adolescent representative.

Non-prioritization of mental health in healthcare settings leaves most mental issues among AYP undiagnosed and unaddressed.

“*…If a young person comes to the facility, they don’t take it as a major problem because you’ll just be treated for your malaria or be treated for your SRH or contraceptives, but if MH is integrated at that point, probably the reason why I’m coming there is not just for contraceptives, but I may be having some other problems or challenges as a young person…*”

Adolescent representative.

Another service provider challenge identified was the lack of adolescent-friendly spaces and customized services for adolescents in most health care settings. The adolescents also indicated that the services are not population-focused—examples including the services provided to teenage mothers.

“*They are all treated as mothers, but besides this young person having had a child, they are still young people, but they are given services just as any other mother would be given without considering the fact that they have other special needs…*”

Adolescent representative.

The AYP expressed more comfort in peer-to-peer mental health services and requested more YACHs to be trained and stationed in health care settings.

“*…involving young people as part of studies. Like, they can be trained the way we train peer educators at facilities, we can have young trained counselors, I don’t know how to describe that because we trust each other, but I’d rather trust you when you have the correct information or you are capable and you have the skills to be able to help me…*”

Adolescent representative.

Other challenges identified within the health care settings included stigma related to mental health due to segregation of mental health service units, lack of confidentiality, inadequate comprehensive assessment tools, and insufficient coordination of mental health services at health facilities, creating a barrier to seeking mental health care.

“*…even public facilities are there, but they are not well integrated, they are separate; the ones that I have seen personally, mental health is separate so it comes with stigma, so when a young person and my friend saw me go that direction, they would ask me whether I’m crazy and ask why do I have to see a counselor because it will look like I have a psychotic problem…we have got providers’ confidentiality or anonymity, this is the set up to the facility…*”

Adolescent representative.

### 3.3. Adolescent Level Needs

Lack of parental/caregiver support and compromised family support structures were identified as critical challenges that predispose AYP to most mental health challenges experienced at this age.

“*…The major challenges were stress and depression, and these are the main problems that adolescents and young people undergo. Stress and depression are caused by society, parents and siblings. Relationships like the family, i.e., the people around you, because they can also impact depression or mental issues on you…*”

Adolescent representative.

Pressure to meet parental and societal expectations was also highlighted as a key precipitant to mental health issues in AYPS.

“*…The society may be judgmental at some point; you know, things are evolving because the things that our grandparents used to do in the 1990s are not the same things in 2021. Things are really much evolving, and maybe the society expects us to behave in such a way that the young generation cannot be able to, and when that pressure is impacted to young people, then automatically it brings some effects or some pressure on us…*”

Adolescent representatives.

Other adolescents reported that preexisting inherent mental health disorders are also some of the challenges they have to deal with.

“*…’Personality disorders’ and … some of the issues that are considered because they contribute factors in low productivity among youth … if you have all this, you are not able to be productive for yourself, your family, and the community that you are in, and then if they are not addressed…*”

Adolescent representative.

The AYP also illustrated that social stress within peer groups and romantic relationships were key drivers to mental health challenges that arise from inability to meet expectations, either at peer or relationship levels. Identity crises or the need to conform to specific peer groups equally lead to mental complications, especially when the AYP struggles to meet the expectations of their peers.

*“…There is peer pressure with a lot of ‘personality disorders’, and you feel as a young person you want to see each other. Maybe you have this boyfriend, and they feel they love them, but when they find out that the boyfriend cannot provide, they end up killing each other or engage in very risky behavior…*”

Adolescent representative.

“*…..and we also have depression, and this is as a result of relationships—there is a lot that is happening in relationships, where we have those peers with a relationship that are working and those that are not working, and so if one engages in an unhealthy or toxic relationship and they are not able to identify the red flags, mostly they end up being depressed because they are trying to balance and to ensure that their relationship works at the same time…*”

Adolescent representative.

Challenges within the digital social networking platforms, such as cyber bullying and unrealistic lifestyles, have also led to mental health problems among AYP.

“*…Another problem is social stress because there is an increase in the use of technology … there is an increase in interaction of young people of different social classes, so that peer pressure that comes from the peers and friends, maybe about achievements that they need and realities…*”

Adolescent representative.

Inability to cope with mental health challenges due to maladaptive coping mechanism skills is also a key challenge among adolescents—most AYP resort to drug and substance abuse, risky practices, and even committing suicide. Existing mental health disorders were also noted to increase the AYPs’ risks of drug and substance abuse and other behavior problems as the young adults attempt to establish coping mechanisms.

“*Even though one can quickly bounce back from some of the challenges experienced by adolescents without progression to severe mental health disorders, most adolescents cannot achieve this because of maladaptive coping skills, which worsen the mental situation. So, there is also lack of proper coping skills … Like most young people are trying to look for other innovative negative ways of coping with mental health issues. Some are using drugs, and some are even trying to do other crazy things so that they can avoid…*”

Adolescent representative.

“*….You will find that some end up in drugs, which also brings about crime because you are not able to sustain the habit you have started, meaning that in the end, you end up becoming a thief or a thug in order to sustain the habit you had started, the suicides have become higher. We have a lot of mental health issues that affect young people, either leading to suicide or attempted suicide, and you can see from many reports that a lot of young people are either involved in killing each other or trying to arm themselves, and this is because of the mental health problems…*”

Adolescent representative.

“*…There are accidents, maybe there is defilement, there are some emotional abuses, and when we suppress these feelings without good coping skills, we will end up having these challenges, so we also see these issues as the most important because they cause a lot of issues that we may not know how to overcome, so most of these issues are that they are not easily recognized, and if young people are not enlightened, they will not be able to recognize them…*”

Adolescent representative.

Adolescents also cited scarce preventive services to control the occurrences and the progression into severe mental disorders. Therefore, young people seek help from non-professionals and unreliable online sites.

“*……..when young people are depressed, you will find them googling how to prevent depression while others google funny things, so there are the most common use sources in the internet; some seek assistance from parents or guardians or siblings or even friends who they can trust, and there are also other community-based organizations which offer programs that support mental health, which are also good places where young people seek help, so we also have those who seek from religious groups and those are pastors, sheikhs, and fathers…….*”

Adolescent representatives.

## 4. Key Stakeholders’ Recommendation to Address Identified Challenges 

**Figure 2:**
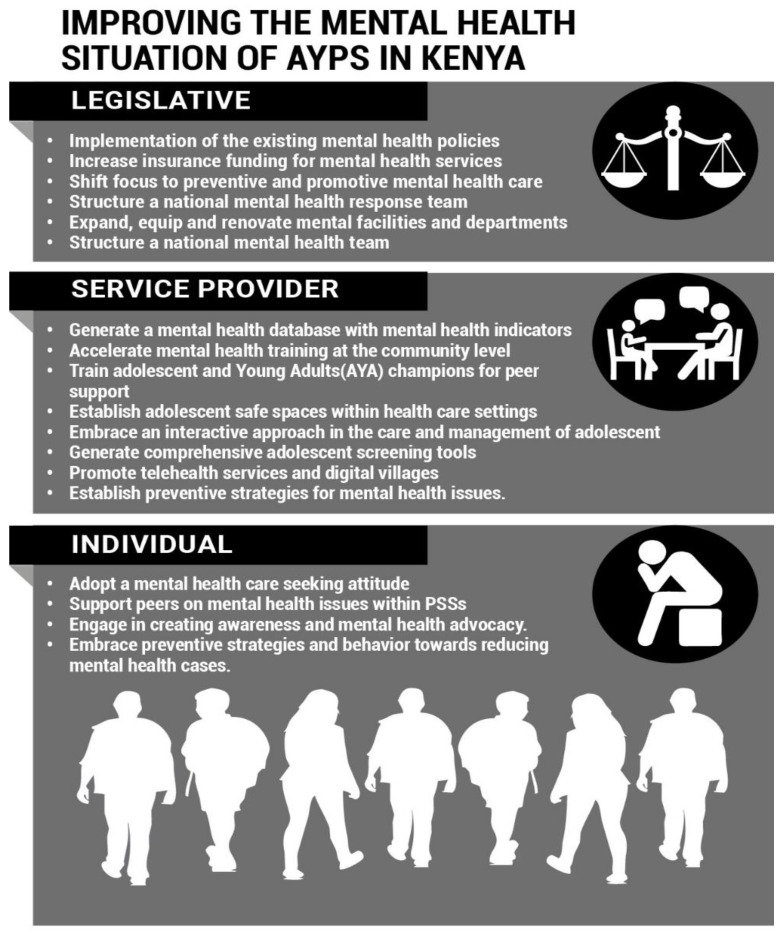
The image illustrates the stakeholders’ recommendations for improving the mental health situation of AYP in Kenya.

The stakeholders identified 11 key recommendations aimed towards addressing the mental health challenges faced by AYPs in Kenya and improving mental health outcomes. These included (1) an increase in insurance financing, (2) the acceleration of community health interventions, (3) the establishment of adolescent-friendly spaces, (4) training of adolescent youth champions, (5) interactive service provision models, (6) implementation of the existing mental health policies and structures, (7) the development of comprehensive assessment tools, (8) well-equipped mental health departments in health facilities, (9) the enhancement of telehealth services and digital villages, (10) the establishment of a functional mental health response team, and (11) the development of a mental health database. The proposals were considered within the same clusters established while identifying the mental health challenges, as indicated in Figure 2.

### 4.1. Legislative Recommendations

The government needs to increase insurance financing, for example, by the National Health Insurance Fund (NHIF) to provide comprehensive coverage, including mental health care patients. The IRA (Insurance Regulatory Authority) can ensure that companies are compliant with the law regarding the issuance of equitable medical cover for all medical health conditions, including substance use disorders and suicide attempts among adolescents and young adults, to improve access to mental health services and utilization of mental health services. The government needs to implement the existing mental health structures and amend policies that do not improve mental health access. An example is the Mental Health Act enacted in 1989, which requires amendments to align with the constitution to advocate for the rights of persons with mental health conditions to be addressed with respect and with dignity and to be accorded equal rights. This step will reduce mental health disparities by curbing the stigma associated with mental health issues and promote mental health-seeking behaviors among AYPs.

The country needs an effective national mental health response team. Experts have projected a rise in mental health cases in the country owing to the effects of the COVID 19 pandemic. The Ministry of Health should consider upscaling psychosocial guidelines established for health workers to promote mental health support for the general population, including AYP, to regulate the mental effects of the pandemic. More mental health facilities are required in every county with well-equipped mental health departments, and dilapidated structures need to be revamped to decentralize safe mental health service delivery environments. The government also needs to provide effective medication for the treatment of mental health disorders. Currently, most public facilities dispense first-line medicines with numerous side effects, affecting the adherence to these drugs because the more effective second-line drugs are costly.

Other considerations include establishing promotive and preventive strategies to enhance early identification and intervention on AYP mental health issues rather than mitigation. Examples include mental health advocacy and the implementation of preventive programs in the AYPs’ primary social environments and primary health care settings.

### 4.2. Service Provider/MoH Recommendations

Through the Division of Community Health, the Ministry of Health can accelerate the training of the Community Health Assistants (CHAs) and Community Health Volunteers (CHVs) with the existing manuals to ensure mental health services at primary levels. The approach will also enhance the collection of data that depict the country’s actual mental health situation, boost referrals, and promote care access for mental health patients. Data form an integral aspect of planning and implementing mental health interventions [22]. Therefore, the MoH should consider developing a reliable mental health database and mental health indicators to capture all mental health cases and reflect the accurate AYP mental health picture in the country.

The movement of mental health experts to all healthcare facilities and AYP-specific provider training should ensure professional and unbiased mental health services that boost the AYPs’ adherence to mental health interventions. Equally, more training is vital for YACHs to promote Peer Psychosocial Support (PSS), bridge the gap between mental health patients and service providers, and ensure population-focused services in health care settings [23,24]. The PSS approach can also be incorporated into other care services such as SRH, SGBV, VMMC, and HIV care and treatment to ensure a comprehensive mental health response. There is a call for safe spaces in health care settings to enhance confidentiality and anonymity for adolescents seeking mental health services to reduce stigma associated with mental health challenges. For example, clinics integrating adolescent services, including voluntary counseling and testing (HIV testing Services), comprehensive care centers, SRH, SGBV, and psychotherapy can offer walk-in and out services without evidence of the kind of benefits sought therein. The MOH also needs to work the policymakers and implementors to address other interconnected factors including health challenges, including disparities in earnings and employment opportunities, violence, and injuries, and stigma associated with mental health problems.

Comprehensive and tailored mental health screening tools are also critical in assessing the needs of adolescents and young adults in the Kenyan context. Most Western evaluation tools, if directly administered, may not accurately capture the mental health situation among AYPs in the country. Some trials also contain English technical terms that a Swahili-speaking adolescent may not comprehend, hence the need to translate different language versions and translations.

Considering the increasing number of adolescents seeking health information on digital platforms, telehealth services and villages need to be promoted to save time in physically seeking care for services offered virtually [25]. Adolescents have equally been noted to be more candid about mental health issues in digital than physical consultations. Technology increases access to mental health information, such as through one-to-one websites containing health information [26]. Developing an interface through which adolescents can anonymously seek help and talk to their peers on mental health issues can also break the barrier to seeking mental health services. Mental health screening services should also be integrated into the health care continuum to promote early identification and intervention for mental health issues and precipitant factors.

### 4.3. Adolescent Level Recommendations

Adolescents need to develop a mental health care-seeking attitude to utilize the existing mental health care structures to improve their well-being and reduce the prevalence of mental health issues among this population. AYP also need to create awareness and advocate for positive mental health outcomes through social platforms to educate their peers on mental health issues and the availability of care services and reduce the stigma associated with seeking mental health care. Such preventive approaches will promote resilience through information on adaptive coping mechanisms and mental health resources, significantly reducing the mental health gaps among adolescents in the country.

## 5. Discussion

Participatory research is critical to integrating inputs often solicited from various stakeholders at the onset of a study. The process enhances the study’s relevance, especially to the stakeholders, and potentially improves the health outcomes [27]. Therefore, the stakeholders need to understand the topic and the relevance of the intervention. In the REACH-AYA study, stakeholders, including AYP, provided input on mental health issues affecting adolescents living in Kenya. Listening to the voices of young adults is critical in designing services that align with the needs of the targeted population. Thus, collaborating with the AYA to promote first-hand experiences in service planning is significant [28]. According to the MOH, over 60% of the Kenyan population comprises AYA aged between 10–24, and the prevalence of mental health issues among this population is at 10.3%, demonstrating the need to provide safe spaces and environments that promote psychosocial support to mitigate the negative mental health outcomes and the need for evidence-based interventions [29]. In the Nyanza region of Kenya, with a high HIV prevalence, adolescents are likely to lack parental/social support networks, and there is a lack of basic resources, elevating the risk of risky behavior and mental disorders [30].

The study builds on the existing body of literature showing that mental challenges among adolescents in Kenya are often underrated, undiagnosed, and untreated, impacting negatively on the health outcomes [30,31]. The study findings underscore that mental health issues among adolescents are precipitated and perpetuated by legislative challenges, health care service gaps, and individual issues. The stakeholders categorized mental health issues among adolescents into three clusters—legislative, health care service gaps, and individual challenges—with each cluster containing specific challenges that affect adolescent mental health, as indicated in Figure 1. The clusters provide a framework for clarifying the issues affecting health, and preventive strategies and interventions are incorporated within the contexts of the clusters.

Figure 2 contains the potential intervention challenges identified in the three clusters. Health care service interventions are critical for improving mental health outcomes in the country. A study conducted among AYP living with HIV in Kenya indicated that AYA-specific service provider training is linked to enhanced AYA engagement. The adequately trained providers are also more likely to adhere to national treatment guidelines and may be equipped with better AYA communication skills [32]. Interventions that enhance socioeconomic stability can also decrease the risks of mental health symptoms among the AYA population. Studies on poverty-targeted unconditional cash transfer programs have been documented to improve mental health outcomes among AYA in underserved regions [33]. A church-based intervention for families to promote mental health and prevent HIV among adolescents in rural Kenya also demonstrated the significance of promoting family and social structures in improving mental health outcomes among adolescents [34].

The participant list provides an insight of the broad background of the stakeholders involved in the study.

## 6. Study Strengths

By listening to the voices of the adolescents, the study provided a first-hand experience that is critical for service planning, reviewing, and coordinating county-level mechanisms.

## 7. Study Limitations

The study relied on the interpretations of the AYA in illustrating the mental health challenges affecting the population. Most of the participants were not mental health experts, increasing the chances of misrepresentation.

## 8. Conclusions

As demonstrated in this study, several gaps exist in the mental health service in Kenya, illustrating the need for immediate action plans that strengthen mental health outcomes among the AYP population. Deliberation with stakeholders, especially the YACH, was integral in assessing and mitigating mental health gaps. By allowing the voice of the target population to address its problems and effect change, the study captured the critical issues among the AYP. The high participation by the AYP indicated that the population appreciated the involvement in policymaking. The study highlights the need for additional adolescent-focused mental health literacy among primary health service providers to enhance accuracy in diagnosing and managing mental health disorders. The research also enhances existing policies and strategies or designs new ones aimed towards more critical and sustainable mental health interventions.

There is a paucity of high-quality data on AYA mental health issues, with most studies focusing on the mental health effects of HIV among the AYA. This study provides knowledge on the impact of mental health issues on the AYA, including those not living with HIV. The study results and methods will serve as an ideal reference and enhance knowledge on adolescent mental health topics. The study will also serve as a basis for evidence-based intervention strategies. Such studies also improve mental health outcomes, since young people extensively use digital platforms to research mental health issues. Further studies will be critical in evaluating the impact of this and other related studies in improving mental health outcomes among the AYA.

## Figures and Tables

**Table 1:** Stakeholders’ demographics.

	Sex		Stakeholder Categorization	
Age	Male	Female	Youth	MoH Officials/Researchers/Program Implementers
21–24 years	5 (36%)	9 (64%)	14 (100%)	0 (0%)
25–29 years	2 (33.3%)	4 (66.7%)	2 (33.3%)	4 (66.7%)
30–34 years	3 (50%)	3 (50%)	0 (0%)	6 (100%)
>35 years	6 (40%)	9 (60%)	0 (0%)	15 (100%)
Total	**16 (39%)**	**25 (61%)**	**16 (39%)**	**25 (61%)**

## Appendix A. List of Contributors

**Table A1:** List of contributors/stakeholders.

Name	Work Station/Affiliation	Country	County/State
Pauline Mutie	YACH-Mombasa	Kenya	Mombasa
Vivian Edwick Ochieng’	YACH-Mombasa	Kenya	Mombasa
Thomas Omache	YACH-Mombasa	Kenya	Mombasa
Elma Atieno	YACH-Kisumu	Kenya	Kisumu
Laura Joy Okatch	YACH-Kisumu	Kenya	Kisumu
Mercy Jane	YACH-Kisumu	Kenya	Kisumu
Gideon Obuya	YACH-NMS	Kenya	Nairobi
Caroline Ngonyo Wanjiku	YACH-NMS	Kenya	Nairobi
Felix Onyango	YACH-NMS	Kenya	Nairobi
Maryann Wanjiru	YACH-NMS	Kenya	Nairobi
Margaret Akinyi Atieno	YACH-NMS	Kenya	Nairobi
Quinter Milly	YACH-NMS	Kenya	Nairobi
Derrick Omondi	YACH-NMS	Kenya	Nairobi
Lilian Kunyiha	LVCT Health	Kenya	Nairobi
Rahab Nancy Mukami	LVCT Health	Kenya	Nairobi
Caroline Obare	LVCT Health	Kenya	Kisumu
Lomlen Stephen	LVCT Health	Kenya	Nairobi
Martha Biyaki	LVCT Health	Kenya	Nairobi
Mercy Nzuki	LVCT Health	Kenya	Nairobi
John Mbugua	LVCT Health	Kenya	Nairobi
Nicholas Otieno Odhiambo	LVCT Health	Kenya	Kisumu
Hannah Muna	LVCT Health	Kenya	Mombasa
Rose Sadah Musinda	LVCT Health	Kenya	Nairobi
Anthony Chazara	LVCT Health	Kenya	Mombasa
Lilian Maina	LVCT Health	Kenya	Nairobi
Gitau Desma	LVCT Health	Kenya	Nairobi
Michelle Oluoch	LVCT Health	Kenya	Nairobi
Denis Osumo	LVCT Health	Kenya	Nairobi
